# Supplementary material for: Future life expectancy in Europe taking into account the impact of smoking, obesity, and alcohol
Source: eLife. 2021 Jul 6;10:e66590. doi: 10.7554/eLife.66590 (PMC8337079; doi:10.7554/eLife.66590)
Supplement: Supplementary file 1. [file elife-66590-supp1.docx]

**Supplementary file 1**

**Supplementary file 1A. Sex differences in observed (2014) and projected (2065) life expectancy at birth (e0), by country and sex, according to our projection methodology, which takes into account the impact of smoking, obesity, and alcohol and the mortality experiences of forerunner countries (“lifestyle and coherent”); the benchmark Lee-Carter extrapolative mortality projection applied to all-cause mortality (“Lee-Carter”); and when purely accounting for smoking, obesity, and alcohol (“adding lifestyle”)**

|  | Sex difference e0 (women minus men) | | | |
| --- | --- | --- | --- | --- |
|  |  | 2065 | | |
| Country | 2014 | Lee-Carter | Adding lifestyle | Lifestyle and coherent |
|  |  |  |  |  |
| Austria | 4.82 | 2.51 | 3.55 | 2.16 |
| Belgium | 4.95 | 2.32 | 4.23 | 2.05 |
| Czech Republic | 6.01 | 3.10 | 5.05 | 2.51 |
| Denmark | 4.10 | 1.94 | 4.85 | 2.58 |
| Finland | 5.73 | 2.65 | 4.36 | 2.47 |
| France | 6.16 | 3.66 | 4.22 | 2.62 |
| Germany | 4.91 | 2.42 | 3.93 | 2.44 |
| Greece | 5.37 | 4.68 | 4.19 | 1.47 |
| Hungary | 6.98 | 5.35 | 4.32 | 2.31 |
| Ireland | 4.07 | 2.08 | 3.80 | 2.45 |
| Italy | 4.61 | 3.05 | 4.19 | 2.10 |
| Netherlands | 3.42 | 0.13 | 3.86 | 2.07 |
| Norway | 4.07 | 1.86 | 3.25 | 2.48 |
| Poland | 7.76 | 5.22 | 4.46 | 2.51 |
| Slovenia | 5.72 | 3.51 | 4.85 | 2.69 |
| Sweden | 3.70 | 1.54 | 3.09 | 2.37 |
| Switzerland | 4.18 | 1.64 | 3.39 | 2.28 |
| United Kingdom | 3.74 | 0.94 | 3.35 | 2.30 |
|  |  |  |  |  |
| *Average* | *5.02* | *2.70* | *4.05* | *2.33* |
| *Min* | *3.42* | *0.13* | *3.09* | *1.47* |
| *Max* | *7.76* | *5.35* | *5.05* | *2.69* |
| *Variance* | *1.35* | *1.85* | *0.31* | *0.08* |

**Supplementary file 1B. Observed (2014) and projected (2065) life expectancy at birth (e0) according to different methodologies*, by country and sex**

|  | **e0 2014** | | **e0 2065** | | | | | |
| --- | --- | --- | --- | --- | --- | --- | --- | --- |
|  | **All-cause mortality** | **Non-lifestyle-attributable mortality** | **All-cause mortality** | | **Non-lifestyle-attributable mortality** | | **Non-lifestyle- + lifestyle-attributable mortality** | |
| **Country** |  |  | **Indiv. LC** | **Coherent** | **Indiv. LC** | **Coherent** | **Indiv. LC** | **Coherent** |
| (1) | (2) | (3) | (4) | (5) | (6) | (7) | (8) | (9) |
| **Men** |  |  |  |  |  |  |  |  |
| Austria | 78.91 | 82.72 | 88.91 | 88.85 | 90.28 | 91.43 | 89.02 | 90.38 |
| Belgium | 78.57 | 82.96 | 87.76 | 88.57 | 89.26 | 91.69 | 88.20 | 90.93 |
| Czech Republic | 75.72 | 80.64 | 87.77 | 86.68 | 88.93 | 90.20 | 87.23 | 88.86 |
| Denmark | 78.56 | 82.44 | 87.72 | 88.49 | 89.53 | 91.43 | 88.48 | 90.50 |
| Finland | 78.13 | 81.92 | 88.25 | 88.36 | 89.37 | 91.15 | 88.30 | 90.22 |
| France | 79.28 | 83.76 | 89.11 | 89.44 | 91.19 | 92.26 | 90.03 | 91.40 |
| Germany | 78.43 | 82.54 | 88.36 | 88.47 | 90.14 | 91.37 | 88.67 | 90.18 |
| Greece | 78.46 | 82.92 | 85.07 | 88.98 | 88.32 | 92.06 | 86.32 | 90.97 |
| Hungary | 72.26 | 80.30 | 81.74 | 84.29 | 87.78 | 90.19 | 85.89 | 88.64 |
| Ireland | 79.15 | 82.66 | 89.59 | 88.99 | 91.34 | 91.70 | 90.57 | 90.95 |
| Italy | 80.55 | 83.83 | 89.25 | 89.63 | 90.20 | 92.19 | 89.14 | 91.43 |
| Netherlands | 79.87 | 83.24 | 88.67 | 89.17 | 89.54 | 91.79 | 88.73 | 91.11 |
| Norway | 80.03 | 82.80 | 88.86 | 89.13 | 90.85 | 91.53 | 90.23 | 90.87 |
| Poland | 73.66 | 80.15 | 85.21 | 85.76 | 89.73 | 90.58 | 88.28 | 89.37 |
| Slovenia | 77.96 | 82.03 | 88.68 | 88.01 | 90.07 | 90.85 | 88.95 | 89.91 |
| Sweden | 80.35 | 82.66 | 88.37 | 89.38 | 89.88 | 91.31 | 89.18 | 90.64 |
| Switzerland | 80.93 | 83.63 | 89.77 | 89.87 | 90.50 | 91.92 | 89.31 | 90.88 |
| United Kingdom | 79.25 | 82.79 | 88.99 | 89.17 | 90.25 | 91.78 | 89.35 | 91.04 |
| *Average* | *78.34* | *82.44* | *87.89* | *88.40* | *89.84* | *91.41* | *88.66* | *90.46* |
| *Min* | *72.26* | *80.15* | *81.74* | *84.29* | *87.78* | *90.19* | *85.89* | *88.64* |
| *Max* | *80.93* | *83.83* | *89.77* | *89.87* | *91.34* | *92.26* | *90.57* | *91.43* |
| *Variance* | *4.98* | *1.12* | *3.77* | *1.96* | *0.79* | *0.36* | *1.41* | *0.62* |
| **Women** |  |  |  |  |  |  |  |  |
| Austria | 83.73 | 85.71 | 91.42 | 91.58 | 93.14 | 93.17 | 92.57 | 92.54 |
| Belgium | 83.52 | 85.86 | 90.09 | 91.51 | 92.98 | 93.51 | 92.43 | 92.98 |
| Czech Republic | 81.73 | 84.06 | 90.87 | 90.05 | 92.81 | 92.01 | 92.28 | 91.37 |
| Denmark | 82.67 | 86.00 | 89.66 | 90.97 | 93.82 | 93.52 | 93.33 | 93.08 |
| Finland | 83.85 | 85.81 | 90.90 | 91.65 | 93.26 | 93.33 | 92.65 | 92.69 |
| France | 85.44 | 87.20 | 92.77 | 92.90 | 94.73 | 94.46 | 94.24 | 94.01 |
| Germany | 83.35 | 85.67 | 90.78 | 91.24 | 93.16 | 93.25 | 92.60 | 92.62 |
| Greece | 83.83 | 85.36 | 89.75 | 91.52 | 91.16 | 93.01 | 90.51 | 92.44 |
| Hungary | 79.24 | 83.07 | 87.09 | 88.73 | 90.89 | 91.67 | 90.21 | 90.96 |
| Ireland | 83.23 | 86.25 | 91.67 | 91.56 | 94.93 | 93.99 | 94.38 | 93.40 |
| Italy | 85.16 | 86.81 | 92.30 | 92.54 | 93.91 | 94.09 | 93.34 | 93.53 |
| Netherlands | 83.29 | 86.13 | 88.80 | 91.30 | 93.03 | 93.58 | 92.59 | 93.18 |
| Norway | 84.09 | 86.58 | 90.72 | 91.71 | 93.87 | 93.75 | 93.48 | 93.35 |
| Poland | 81.41 | 84.25 | 90.44 | 90.40 | 93.41 | 92.65 | 92.74 | 91.88 |
| Slovenia | 83.69 | 85.71 | 92.20 | 91.42 | 94.23 | 93.15 | 93.79 | 92.60 |
| Sweden | 84.05 | 86.19 | 89.92 | 91.67 | 92.68 | 93.40 | 92.27 | 93.01 |
| Switzerland | 85.11 | 86.76 | 91.41 | 92.23 | 93.19 | 93.67 | 92.70 | 93.16 |
| United Kingdom | 82.99 | 86.08 | 89.93 | 91.40 | 93.21 | 93.80 | 92.70 | 93.34 |
| *Average* | *83.35* | *85.75* | *90.59* | *91.35* | *93.25* | *93.33* | *92.71* | *92.78* |
| *Min* | *79.24* | *83.07* | *87.09* | *88.73* | *90.89* | *91.67* | *90.21* | *90.96* |
| *Max* | *85.44* | *87.20* | *92.77* | *92.90* | *94.93* | *94.46* | *94.38* | *94.01* |
| *Variance* | *2.02* | *1.01* | *1.73* | *0.81* | *0.99* | *0.45* | *1.07* | *0.55* |

* We compared our projection approach (which comprises the coherent Li-Lee projection of non-lifestyle-attributable mortality, added with projected lifestyle-attributable mortality)(column 9), with a Lee-Carter projection of all-cause mortality (column 4), and of non-lifestyle-attributable mortality (column 6). Also, we employed a coherent Li-Lee projection of all-cause mortality (column 5) and of non-lifestyle attributable mortality (column 7). In addition, we assessed the effect of purely adding lifestyle to a Lee-Carter projection (column 8).

**Supplementary file 1C. Comparison of projected life expectancy in 2065 between our final projection (“we”) and the most recent projections by Eurostat and the United Nations (UN), by country and sex**

|  | Men | | | Women | | | Women minus Men | | |
| --- | --- | --- | --- | --- | --- | --- | --- | --- | --- |
|  | We | Eurostat | UN | We | Eurostat | UN | We | Eurostat | UN |
| Austria | 90.4 | 85.7 | 85.7 | 92.5 | 89.7 | 89.2 | 2.2 | 4.0 | 3.5 |
| Belgium | 90.9 | 85.8 | 85.8 | 93.0 | 89.8 | 90.9 | 2.1 | 4.0 | 5.1 |
| Czech Republic | 88.9 | 84.1 | 84.0 | 91.4 | 88.7 | 85.4 | 2.5 | 4.6 | 1.4 |
| Denmark | 90.5 | 85.5 | 85.5 | 93.1 | 89.2 | 87.7 | 2.6 | 3.7 | 2.2 |
| Finland | 90.2 | 85.6 | 85.3 | 92.7 | 89.9 | 89.7 | 2.5 | 4.3 | 4.3 |
| France | 91.4 | 86.1 | 85.5 | 94.0 | 91.0 | 89.0 | 2.6 | 4.9 | 3.5 |
| Germany | 90.2 | 85.4 | 85.5 | 92.6 | 89.4 | 89.7 | 2.4 | 4.0 | 4.2 |
| Greece | 91.0 | 85.8 | 86.0 | 92.4 | 89.8 | 91.0 | 1.5 | 4.0 | 5.0 |
| Hungary | 88.6 | 82.7 | 81.1 | 91.0 | 87.8 | 87.9 | 2.3 | 5.1 | 6.8 |
| Ireland | 90.9 | 86.2 | 86.9 | 93.4 | 89.9 | 84.8 | 2.4 | 3.7 | -2.0 |
| Italy | 91.4 | 86.5 | 87.1 | 93.5 | 90.5 | 89.6 | 2.1 | 4.0 | 2.5 |
| Netherlands | 91.1 | 86.0 | 86.6 | 93.2 | 89.3 | 90.9 | 2.1 | 3.3 | 4.3 |
| Norway | 90.9 | 86.4 | 86.5 | 93.3 | 89.8 | 89.8 | 2.5 | 3.4 | 3.3 |
| Poland | 89.4 | 83.4 | 82.6 | 91.9 | 88.9 | 81.4 | 2.5 | 5.5 | -1.2 |
| Slovenia | 89.9 | 85.2 | 85.2 | 92.6 | 89.9 | 91.7 | 2.7 | 4.7 | 6.5 |
| Sweden | 90.6 | 86.3 | 86.9 | 93.0 | 89.8 | 88.3 | 2.4 | 3.5 | 1.4 |
| Switzerland | 90.9 | 86.9 | 87.5 | 93.2 | 90.5 | 87.0 | 2.3 | 3.6 | -0.4 |
| United Kingdom | 91.0 | NA | 86.0 | 93.3 | NA | 90.2 | 2.3 | NA | 4.2 |
|  |  |  |  |  |  |  |  |  |  |
| *Average* | *90.5* | *NA* | *85.5* | *92.8* | *NA* | *88.6* | *2.3* | *NA* | *3.0* |
| *Average (not UK)* | *90.4* | *85.5* | *85.5* | *92.8* | *89.6* | *88.5* | *2.3* | *4.1* | *3.0* |
| *Min* | *88.6* | *82.7* | *81.1* | *91.0* | *87.8* | *81.4* | *1.5* | *3.3* | *-2.0* |
| *Max* | *91.4* | *86.9* | *87.5* | *94.0* | *91.0* | *91.7* | *2.7* | *5.5* | *6.8* |
| *Variance* | *0.6* | *NA* | *2.4* | *0.6* | *NA* | *6.3* | *0.1* | *NA* | *5.7* |
| *Variance (not UK)* | *0.6* | *1.2* | *2.5* | *0.6* | *0.5* | *6.5* | *0.1* | *0.4* | *5.9* |

Sources: own projections, Eurostat (2020a), United Nations (2020)
